# An innovative inpatient protocol for alcohol withdrawal prevention in a 16-year-old adolescent: a case report

**DOI:** 10.1186/s13256-023-03863-8

**Published:** 2023-04-19

**Authors:** Dina Moubayed, Nicholas Chadi

**Affiliations:** 1 Division of Adolescent Medicine, Department of Pediatrics, Sainte-Justine University Hospital Center, 3175 Chemin de La Côte-Sainte-Catherine, Montreal, QC H3T 1C5 Canada; 2 Faculty of Medicine, Université de Montréal, Montreal, QC Canada

**Keywords:** Adolescent, Alcohol, Protocol, Substance use disorder, Withdrawal, Case report

## Abstract

**Background:**

Alcohol cessation in youth with daily drinking poses a risk of severe and life-threatening alcohol withdrawal. If unsupervised, alcohol withdrawal in heavy users can cause severe complications, such as seizures, delirium tremens, and death. We present the case of a teenager admitted at our pediatric center for the prevention of alcohol withdrawal using an innovative protocol, including a fixed-dosage benzodiazepine regimen.

**Case description:**

A 16-year-old Caucasian male, known to have anxiety and an attention deficit disorder, was electively admitted for medical stabilization and surveillance of alcohol withdrawal. He had been previously diagnosed with alcohol use disorder and had a past history of withdrawal symptoms. He was prescribed a course of thiamine, folic acid, as well as a fixed-dosage benzodiazepine taper over 5 days. His withdrawal symptoms were evaluated using a standardized Clinical Institute Withdrawal Assessment for Alcohol scale. During his stay, he reported minimal symptoms, as well as a score on the Clinical Institute Withdrawal Assessment for Alcohol scale consistently lower than 5. His mood, motivation, eating habits and sleeping patterns significantly improved during his stay. He developed no medical complications and demonstrated pride in his successes. He was successfully transferred to a long-term rehabilitation center.

**Conclusions:**

A withdrawal prevention protocol was developed on the basis of existing literature. It included a soothing environment, basic laboratory work evaluating the medical complications of alcohol use, as well as medication aiming to prevent and reduce potential withdrawal symptoms. The patient responded well to the fixed-dosage taper with minimal symptoms and discomfort. Although alcohol use in adolescents is frequent, alcohol withdrawal in this population is rarely seen in a pediatric hospital setting. Nonetheless, given the lack of existing guidelines regarding alcohol withdrawal in adolescents, standardized protocols could be greatly beneficial for the prevention of this condition in this population.

## Background

In most countries, the average age for alcohol use onset is considerably lower than legal drinking age [1]. Moreover, by the age of 16, approximately one-third of adolescents in North America report drinking at least 2 times per week [2, 3]. Though rates of alcohol use and binge drinking among adolescents have been trending downwards in the past three decades [1], alcohol use disorder should still be considered a pediatric onset disease, with high levels of substance use and mental health comorbidities.

In youth with daily drinking, alcohol cessation poses a risk of alcohol withdrawal, which can lead to severe complications such as seizures, delirium tremens, and even death [4]. In adult settings, alcohol withdrawal protocols have been developed for inpatient and outpatient treatment, on the basis of the severity of withdrawal symptoms [5]. Currently, very few pediatric centers offer standardized inpatient medical prevention and treatment for alcohol withdrawal, which may place youths’ health at risk. We present the case of an adolescent admitted for prevention and potential treatment of alcohol withdrawal in a tertiary pediatric center.

## Case description

A Caucasian 16-year-old male was electively admitted for medical stabilization and surveillance of alcohol withdrawal. His past medical history was significant for attention deficit disorder with hyperactivity, oppositional defiant disorder, anxiety disorder, insomnia, and a single episode of substance-induced psychosis after using cannabis. His family history was significant for alcohol use disorder (father, mother, and grandparents). Upon admission, he was taking citalopram 40 mg daily and quetiapine 25 mg as needed for sleep. He was prescribed a course of lisdexamfetamine, which he was not currently taking. There had been prior involvement of child protective services because of school absenteeism. A community youth addiction counselor trained in social work had recently started working with the adolescent and was providing weekly home visits, which prompted a consultation at the adolescent substance use clinic at our tertiary care pediatric hospital.

During the initial assessment, the patient reported having his first drink at age 11. In the 3 years prior to presentation, his alcohol consumption had increased from occasional to daily, which had been maintained in the past 12  months. Past-year drinking patterns included a minimum of 6 regular-sized beers daily (including at least 24 beers every weekend) and a few shots of hard liquor per week. There was a significant decrease in functioning at that time, and the patient had stopped attending school since turning 16. His sleeping patterns also changed drastically from nocturnal to diurnal. He had tried to quit drinking twice on his own prior to presentation, both times for a period of less than 24 hours, but had reported withdrawal symptoms, such as tremors, anxiety, and diaphoresis. The patient also used 1 pack of cigarettes per day. He denied current use of other substances. He received a diagnosis of severe alcohol use disorder (AUD) and nicotine use disorder, as per Diagnostic and Statistical Manual of Mental Disorders (DSM-5) criteria. An elective admission for supervised alcohol withdrawal prevention and potential treatment was organized in the following month to help the patient quit drinking in a safe and supervised setting.

Upon admission, the patient reported that his last drinking episode was 12 hours earlier, in the form of “binge drinking,” and was not quantifiable at that time. Physical examination, including assessment of vital signs, was within normal limits and collaboration was adequate, although he appeared highly anxious. His weight was 79.4 kg, and his height was 172 cm. The patient’s readiness to change and personal goals were assessed using motivational interviewing techniques. He demonstrated strong motivation for alcohol cessation, being in the action stage of change, following Prochaska’s stages of change model [6]. Abstinence from alcohol use was discussed, but a harm reduction approach was preferred, to support the formation of a strong therapeutic alliance and encourage adherence to treatment. The patient expressed that his goals were to reduce his alcohol consumption to weekends only, attend school, avoid youth protection services, and return to normal functioning with family and friends.

**Fig. 1:**
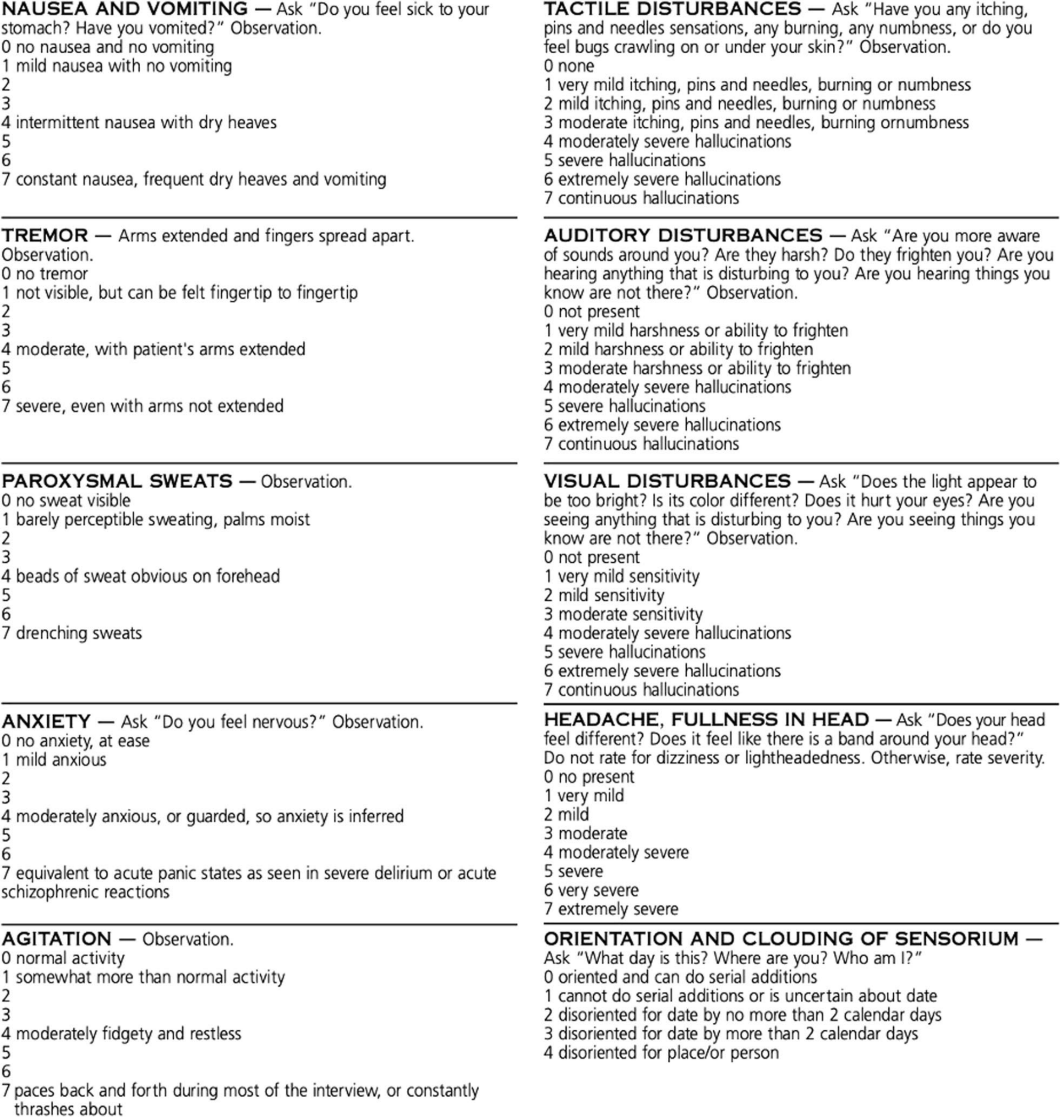
Alcohol withdrawal assessment scoring guidelines (CIWA-Ar). Mild CIWA-Ar < 10, moderate CIWA-Ar 10–18, severe CIWA-Ar > 19. The CIWA-Ar is not copyrighted and may be reproduced freely. Reproduced from Sullivan, J.T.; Sykora, K.; Schneiderman, J.; Naranjo, C.A.; and Sellers, E.M. Assessment of alcohol withdrawal: The revised Clinical Institute Withdrawal Assessment for Alcohol scale (CIWA-Ar). British Journal of Addiction 84:1353–1357, 1989

An individualized pediatric inpatient withdrawal prevention protocol was developed on the basis of a review of the literature and adapted from existing pediatric and adult alcohol withdrawal protocols (see Table 1) [4, 7–10]. It included a soothing environment and basic laboratory work, screening for medical complications of alcohol use, such as liver enzymes levels, coagulation studies, a complete blood count, creatine kinase levels, and thyroid stimulating hormone levels, which were all within normal range. A urine drug screen was negative, as were blood ethanol and other alcohol levels. An electrocardiogram was performed to monitor corrected QT interval, which was normal. Medication aiming to prevent and reduce withdrawal symptoms was prescribed. The patient received a course of thiamine 100 mg three times daily, folic acid 1 mg once daily, ondansetron 8 mg every 8 hours as needed, and pantoprazole 40 mg daily. He was also prescribed melatonin 3 mg every night for insomnia and nicotine replacement therapy (21 mg patch once daily and 2 mg gum every 1–2 hours as needed) to address nicotine withdrawal. The patient was then prescribed a fixed-dosage benzodiazepine taper over 6 days (Table 2). Withdrawal symptoms were evaluated four times daily (and two additional times during the first night of the patient’s stay) using the standardized revised Clinical Institute Withdrawal Assessment for Alcohol scale (CIWA-Ar; Fig. 1). Upon admission, the patient’s CIWA-Ar score was 7 (low range), which was the highest level reached during the hospital stay. Safety measures, including potential adjustments of the fixed-dosage benzodiazepine taper based on CIWA-Ar score, were planned, but not required.

**Table 1:** Adapted pediatric alcohol withdrawal protocol

Objective	Intervention
Environment	Quiet room, soothing environment, time and space orientation reminders (e.g., clocks, room number)
Surveillance	Vital signs and neurological surveillance every 4 hours CIWA-Ar evaluation every 4 hours
Diet	No restrictions, maintain good hydration
Sleep	Melatonin 3–10 mg po at bedtime as needed
Nausea	Ondansetron 8 mg every 8 hours as needed Pantoprazole 40 mg every day
Nicotine replacement therapy	Nicotine patch, 7–21 mg daily as needed Nicotine gums (2–4 mg) or lozenges (1–2 mg) every 1–2 hours as needed
Vitamin supplementation	Thiamine 100 mg three times daily for 1 week, then twice daily for 2 months Folic acid 1 mg every day
Laboratory investigations	• Liver function tests • Coagulation panel • Complete blood count • Creatinine kinase dosage • Electrolyte dosage • Urine drug screening (variable according to center) • Blood alcohol level
Paraclinical investigation	Electrocardiogram
Fixed-dosage benzodiazepine taper with diazepam	• 10 mg four times daily on day 1 • 10 mg three times daily on day 2 • 10 mg twice daily on day 3 • 10 mg daily, at nighttime on day 4 and 5

*CIWA-Ar* Clinical Institute Withdrawal Assessment of Alcohol Scale, revised

**Table 2:** Patient CIWA-Ar scores during the fixed-dosage benzodiazepine taper protocol

	Day 1	Day 2	Day 3	Day 4	Day 5	Day 6
Midnight		1				
4 a.m.		1				
9 a.m.		3	0	2	1	1
Noon		1	0	5	1	1
4 p.m.	7	0	3	3	1	3
9 p.m.	5	0	0	3	4	3
Diazepam taper	10 mg × 1	10 mg four times daily	10 mg three times daily	10 mg twice daily	10 mg at bedtime	10 mg at bedtime

*CIWA-Ar* Clinical Institute Withdrawal Assessment of Alcohol Scale, revised

During his stay, the patient reported minimal withdrawal symptoms and had CIWA-Ar scores that were consistently under 5 (Table 2). He presented few physical symptoms, such as a slight tremor and mild headaches on day 2 of his admission, which disappeared rapidly after a few hours without intervention. His vital signs were stable throughout his stay. He complained of elevated levels of anxiety on his first day. The patient’s primary concern was nicotine cravings and withdrawal symptoms, which were controlled with nicotine replacement therapy. Mood, motivation, appetite, and sleep significantly improved during his stay. He developed no medical complications and was successfully transferred to a long-term rehabilitation center immediately after his hospital stay for a 2-month stay before returning home with his parents. At 10 months post discharge from the hospital, the patient reported drinking only once a week, 5 or 6 beers per occasion on Fridays or Saturdays, and no other substance use except occasional cigarette use (fewer than five per week) and nicotine vaping (approximately one to two 2 mL 5% concentration vaping pods per week). He had resumed normal functioning with family and friends, was working full time at a grocery store, and had plans to return to school and complete his high school degree. He also continued to receive support from his youth community addiction counselor in the form of weekly phone calls or home visits.

## Discussion

Although trends in alcohol use and binge drinking in teenagers are well documented, the incidence of AUD in adolescents is not [11]. In contrast, the consequences of an AUD during this critical developmental period are well established. AUD is associated with an increase in risky behaviors, such as driving under the influence of alcohol and at-risk sexual behaviors, as well as higher rates of sexual assault, violence, school dropout, and severe AUD in adulthood [12]. This adolescent’s case is of particular interest because of the quantity and regularity of alcohol consumed and the severity of the AUD at a young age. The patient admitted to drinking more than 5 times the upper limit of safe alcohol consumption established by the Canadian Center for Substance Use and Addiction [13], which were developed for an adult population. The adolescent’s drinking habits, from a young age, were also associated with a significant decrease in functioning in the patient’s regular activities. Self-medication for an underlying and poorly controlled anxiety disorder was also suspected in this patient. A growing body of literature describes self-medication as a coping strategy used to address the negative feelings associated with mood and anxiety disorders, which may contribute to their high level of comorbidity with substance use disorders as seen in this case [14].

The most common pattern of adolescent drinking being binge drinking, as opposed to regular daily drinking, may explain why intoxication, rather than withdrawal, is more commonly encountered by pediatric healthcare providers. Common signs and symptoms of acute intoxication include ataxia and slurred speech, loss of inhibition, and altered behavior, mood and cognition [10]. However, in our case, the chronic, regular, and significant pattern of use placed the patient at risk of severe alcohol withdrawal syndrome.

Major changes in brain plasticity and neuronal connectivity during adolescence render the adolescent brain highly vulnerable to the long-term effects of alcohol. As such, regular alcohol use during adolescence has been associated with significant neurocognitive changes (working memory loss, lower cognitive functioning) and structural changes, such as smaller grey-white matter volume in the prefrontal and frontal cortex, and cerebellar and hippocampal changes [15, 16].

Alcohol exposure activates the GABAergic system, which causes central nervous system depression. Chronic exposure can lead to down-regulation of GABA receptors and sensitivity, creating an acute imbalance with abrupt cessation of alcohol consumption, leading to withdrawal symptoms [8]. Withdrawal symptoms in adolescents have been associated with poorer neurocognitive performance and may be more harmful for brain functioning and development than the actual intake of alcohol itself [15]. Alcohol withdrawal syndrome begins 6 to 24 hours after the last alcoholic drink and may begin even earlier in teens who have a higher tolerance to alcohol. The severity of alcohol withdrawal is wide-ranged, but its most severe form, delirium tremens, can be life-threatening, leading to seizures and death at its peak (24 to 48 hours). It is uncommon for adolescents to present with severe alcohol withdrawal; however, clinicians should consider severe alcohol withdrawal a medical emergency [10].

Many protocols and guidelines have been developed for alcohol withdrawal in adult care, but clear guidelines are scarce for pediatric care [9, 10]. Based on a review of literature and on provincial guidelines for alcohol withdrawal in adult settings, an individualized protocol was developed. In an adult setting, validated tools to guide the choice of ambulatory versus inpatient care for elective alcohol withdrawal have been developed, but they have not been adapted to the pediatric setting [9]. These tools are based on risk factors of severe alcohol withdrawal, such as: history of severe alcohol withdrawal symptoms on previous quit attempts (such as seizures or delirium tremens), medical comorbidity, tachycardia on presentation, long duration of heavy and regular alcohol intake, and electrolyte abnormalities [9]. Because this was the first case of supervised withdrawal prevention for severe AUD at our center, inpatient admission was chosen for enhanced safety and monitoring.

Benzodiazepines, specifically diazepam, are the gold standard of pharmacological support in alcohol withdrawal, given their strong GABA-mediated effect. While mild withdrawal (CIWA-Ar < 10) does not necessarily require pharmacological support, diazepam was prescribed to prevent withdrawal symptoms. Symptom-triggered therapy or fixed-dosage therapy are two methods that have been widely accepted in the adult literature. Fixed-dosage therapy was chosen in our case to facilitate medication administration and simplify medical and nursing care, given that healthcare workers involved in the case were not familiar with inpatient alcohol withdrawal protocols. Protocols vary in terms of dosage, but the most common protocol found in adult and pediatric guidelines suggest a 10 mg dose of diazepam four to five times a day, with a maximum of 50 mg per day, with a gradual taper over 4 to 5 days [8–10]. The patient responded very well to this dosing regimen, having CIWA-Ar scores consistently under 8, with few symptoms, and did not report over-sedation, which could be an important side effect of benzodiazepine administration.

The CIWA-Ar scale can be used to monitor the course and severity of alcohol withdrawal symptoms (Fig. 1), and was performed every 4 hours, except at nighttime after the first night (to respect the patient’s sleep). In our case, the patient had low scores (< 8) on the CIWA-Ar scale, which indicated mild withdrawal symptoms during treatment. Low scores in the initial stages of alcohol cessation have been associated with little to no risk of severe withdrawal. Of note, high levels of anxiety may cause falsely elevated CIWA-Ar scores, which was suspected on the first day of our patient’s hospital stay (Table 2) [8–10].

Polysubstance use should also be assessed in the planning of elective alcohol withdrawal, and withdrawal from all substances should be treated and monitored accordingly, to limit the development of further incapacitating symptoms [9]. In our case, nicotine withdrawal was present and was responsible for some discomfort expressed by the patient. Nicotine replacement therapy was offered, with satisfactory results. While hospitalized, it is important to maintain a reassuring, quiet environment without excessive stimuli, as it can reduce anxiety and severity of symptoms [8]. The laboratory testing and paraclinical examination performed in our case were on the basis of common recommendations found in guidelines from the adult literature. Blood alcohol level is important to perform, as it can help predict the severity and risk of severe withdrawal symptoms [8, 9]. Vitamin supplementation, primarily thiamine, to prevent Wernicke’s encephalopathy, is widely suggested in the literature and was included in our protocol. Supportive management with pharmacological treatment, such as melatonin for sleep, and dimenhydrinate or ondansetron for nausea, is also recommended to increase patient comfort. As per local guidelines, folic acid was added in our protocol to compensate for the potential low levels of this nutrient in the patient’s diet.

Altogether, the feasibility and acceptability of our protocol was demonstrated by the absence of complications, high levels of patient satisfaction, as well as high acceptability among staff members who were unfamiliar with alcohol withdrawal. The patient’s positive clinical course and return to baseline levels of functioning suggest that our protocol, combined with the care received in rehabilitation and during follow-up, was highly successful in achieving long-term health and harm reduction goals.

## Conclusion

Although adolescent alcohol use is common, alcohol withdrawal is rarely seen in pediatric hospitals. Standardized protocols for the prevention and treatment of alcohol withdrawal have the potential of increasing pediatricians’ level of comfort in providing effective medical care for this condition. Adult protocols can be adapted to pediatric settings, based on adolescents’ needs and goals, to promote a progressive return to baseline levels of functioning. In our case, a fixed-dosage regimen was chosen to prevent withdrawal symptoms and was well tolerated by the patient. To date, there are few services adapted and accessible to teenagers with AUD, and much potential for integration of such adapted services within existing youth preventive, medical, mental health, and dedicated substance use services. There is a need for further research to determine optimal treatment strategies that cater to the unique needs of adolescents with AUD.

## Data Availability

Not applicable.

## Abbreviation

AUD: Alcohol use disorder
CIWA-Ar: Clinical Institute Withdrawal Assessment of Alcohol Scale, revised
